# Impact of a Telemedicine System on Work Burden and Mental Health of Healthcare Providers Working with COVID-19: A Multicenter Pre-Post Prospective Study

**DOI:** 10.1093/jamiaopen/ooac037

**Published:** 2022-05-20

**Authors:** Nobuyuki Kagiyama, Takayuki Komatsu, Masanori Nishikawa, Makoto Hiki, Mariko Kobayashi, Wataru Matsuzawa, Hiroyuki Daida, Tohru Minamino, Toshio Naito, Manabu Sugita, Kunihisa Miyazaki, Hideaki Anan, Takatoshi Kasai

**Affiliations:** 1 Department of Digital Health and Telemedicine R&D, Juntendo University, Tokyo, Japan; 2 Department of Cardiovascular Biology and Medicine, Juntendo University Faculty of Medicine, Tokyo, Japan; 3 Department of Emergency and Critical Care Medicine, Juntendo University Nerima Hospital, Tokyo, Japan; 4 Department of Respiratory Medicine, Fujisawa City Hospital, Fujisawa, Japan; 5 Department of Emergency Medicine, Juntendo University, Tokyo, Japan; 6 Ogino Memorial Laboratory, Nihon Kohden Corporation, Tokyo, Japan; 7 Department of Cardiovascular Biology and Medicine, Juntendo University Graduate School of Medicine; 8 Department of General Medicine, Juntendo University Faculty of Medicine; 9 Department of Surgery, Tokyo-Kita Medical Center, Tokyo, Japan; 10 Emergency Medical Center, Fujisawa City Hospital, Fujisawa, Kanagawa, Japan; 11 Cardiovascular Respiratory Sleep Medicine, Juntendo University Graduate School of Medicine, Tokyo, Japan

**Keywords:** telemedicine, COVID-19, information and communication technology, non-contact monitoring, respiratory monitoring

## Abstract

**Background:**

The Coronavirus disease 2019 (COVID-19) pandemic impacts not only patients but also healthcare providers. This study seeks to investigate whether a telemedicine system reduces physical contact in addressing the COVID-19 pandemic and mitigates nurses’ distress and depression.

**Methods:**

Patients hospitalized with COVID-19 in four hospitals and one designated accommodation measured and uploaded their vital signs to secure cloud storage for remote monitoring. Additionally, a mat-type sensor placed under the bed monitored the patients’ respiratory rates. Using the pre-post prospective design, visit counts and health care providers’ mental health were assessed before and after the system was introduced.

**Results:**

A total of 100 nurses participated in the study. We counted the daily visits for 48 and 69 patients with and without using the telemedicine system. The average patient visits were significantly less with the system (16.3 [5.5–20.3] vs. 7.5 [4.5–17.5] times/day, p = 0.009). Specifically, the visit count for each vital sign assessment was about half with the telemedicine system (all p < 0.0001). Most nurses responded that the system was easy to use (87.1%), reduced work burden (75.2%), made them feel relieved (74.3%), and was effective in reducing the infection risk in hospitals (79.1%) and nursing accommodations (95.0%). Distress assessed by IES-R and depression by PHQ-9 were at their minimum even without the system and did not show any significant difference with the system (p = 0.72 and 0.57, respectively).

**Conclusions:**

Telemedicine-based self-assessment of vital signs reduces nurses’ physical contact with COVID-19 patients. Most nurses responded that the system is easy and effective in reducing healthcare providers’ infection risk.

**Lay Summary:**

In this multicenter pre-post prospective study, we investigated whether a telemedicine system reduces physical contact in addressing the coronavirus disease 2019 (COVID-19) pandemic and mitigates nurses’ mental stress. We counted the daily patient visits before and after using a telemedicine system, with which patients uploaded their vital signs for remote monitoring. Simultaneously, a mat-type sensor for monitoring respiratory rates was also introduced. The average patient visits were less for 69 patients treated with the system than 48 treated without it (16.3 vs. 7.5 times/day, p = 0.009). Specifically, the visit count for each vital sign assessment was about half (all p < 0.0001). A total of 100 nurses answered to a questionnaire and many responded that the system was easy to use (87.1%), reduced work burden (75.2%), made them feel relieved (74.3%), and was effective in reducing the infection risk in hospitals (79.1%) and nursing accommodations (95.0%). Distress assessed by IES-R and depression by PHQ-9 were at their minimum even without the system and did not change with the system (p = 0.72 and 0.57, respectively). Thus, the telemedicine system reduced physical contacts and nurses had favorable impression to the system despite no significant improvement in mental stress scores.

## Introduction

The Coronavirus disease 2019 (COVID-19) has caused a worldwide pandemic. Thus far, over 194 million people have been infected and 4.1 million people have died from the disease (1). Despite the emergence of highly effective vaccines, the pandemic is still ongoing. More than 4,00,000 new cases were reported every day as of the end of July 2021(1). Elderly and vulnerable people are not the only ones at risk of developing a fatal condition through this highly infectious disease. Young and healthy individuals who do not have chronic comorbidities are also at risk (2-4). Additionally, studies have reported that some patients have persistent symptomatic or asymptomatic complications such as fatigue, dyspnea, and evidence of myocarditis. These complications are observed in a wide range of ages (5, 6). Thus, it is imperative to protect healthcare providers working with COVID-19 patients from infection. COVID-19 may be passed on through respiratory droplets, contact routes, and possibly aerosol particles (7-9). Therefore, standard precautions and using personal protective equipment (PPE) are mandatory in taking care of COVID-19 patients (10). During this unprecedented pandemic, healthcare providers are under extreme stress due to the risk of infection. They likewise have extra work burden, such as the frequent donning and doffing PPEs (11-14).

Vital sign measurements are one of the most frequent procedures that require physical contact between healthcare providers and patients. We have previously reported that a telemedicine-based self-assessment of vital signs is feasible and has the potential of replacing physical vital sign measurements by healthcare providers (15). This present study further investigates whether the telemedicine system reduces the physical contact between COVID-19 patients and healthcare providers and mitigates distress and depression.

## Methods and Materials

### *Study Setting*

This study is a multicenter pre-post prospective observation involving four hospitals (two university hospitals and two general hospitals) and one designated accommodation for COVID-19 patients by the municipality. This accommodation serves to quarantine patients who do not require in-hospital care. Patients with dementia, those admitted to intensive care units, and those unable to measure their own blood pressure using a manometer were excluded from the study. The system's details are reported elsewhere (15). Briefly, the self-assessment of vital signs using this system was able to obtain very similar results to the measurements by professional healthcare providers (interclass coefficient correlations 0.83

– 0.92, p<0.001 for all vital signs) without significant time delay. The patients measured their vital signs and uploaded the data to a secured cloud system using the following devices after a 10-minute lecture on the system's usage: a commercially available digital manometer (UA-651BLE: A&D Medical, Tokyo, Japan), a digital thermometer (C217: TERUMO, Tokyo, Japan), a pulse oximeter (SP2; TERUMO, Tokyo, Japan), and the LAVITA gateway (Nihon Kohden, Tokyo, Japan). Only authorized healthcare providers could check and refer to the uploaded data. A mat-type air pressure sensor placed under the bed mattress (Kaigolog Med: Liquid Design Systems Inc., Tokyo, Japan) automatically and continuously measured the patients' respiratory rates. The sensor continuously analyzed subtle changes in the pressure due to respiratory motions to calculate respiratory rates. The acquired data were then automatically uploaded through an iPad application. The systems uploaded data using Wi-Fi networks. Figure 1 summarizes the system's overall pipeline. The feasibility and accuracy of the system were validated in past studies (Figure 1) (15).

The study protocol was approved by the institutional review board of each hospital except for the accommodation, where the protocol was reviewed and approved by the Juntendo University's Research Ethics Committee with the approval number 20-102. Since we did not collect any patient information including patient characteristics in this study, informed consent from patients was waived.

***Per-patient daily visit counts***

We counted the daily visits to each patient room and assessed healthcare providers' distress and depression status before and after the system was introduced in each institute to assess the system's impact on the number of patient room visits and the healthcare providers' mental status. In addition to the total visit counts, we also counted visits specifically for each vital sign check. Visit counts for the admission day and the discharge day were removed from the analysis because the admission and discharge timing (in the morning or at night) directly influences the times of visits. It likewise varies from patient to patient. Even without the system, vital sign measurements were not taken by healthcare providers in the designated accommodation. Thus, this institute was removed from the analysis.

***Per-healthcare provider mental health assessment***

A web-based questionnaire system using Impact of Event Scale-Revised (IES-R) and Patient Health Questionnaire-9 (PHQ-9) assessed nurses' distress and depression status in an anonymized manner (16, 17). Additionally, the nurses were asked how much they agree with the following questions using a visual analog scale: "1. The system is easy to use, 2. The system reduces the work burden, 3. The system makes them feel relieved, 4. The system is effective for reducing the risk for infection to healthcare providers in hospitals, and 5. The system is effective for reducing the risk for infection to healthcare providers in nursing accommodations.” In the visual analogue scale, 0 was “I don’t think so at all”, and 100 was “I completely agree with the question”. A visual analogue scale scores greater than 50/100 was considered favorable.

***Statistical analysis***

The data are presented as median (1$^{st}$ and 3$^{rd}$ quartiles) for continuous variables and as frequency (%) for categorical variables. The Wilcoxon rank sum test was used to test the differences in the visit counts without and with the system. The paired Wilcoxon signed rank test assessed the change in mental health status before and after the system was introduced. All statistical analyses were performed with R version 4.0.2 (The R Foundation for Statistical Computing, Vienna, Austria) with the package ‘exactRankTests’ (REF). A two-tailed p value of $<0.05$ indicated statistical significance.

## Results

### *Visit counts*

We counted the visits for 164 patients (78 without and 86 with the system). Average hospital lengths were not significantly different between two periods (5 [4 – 7] and 5 [4 – 7] days without and with the system, respectively, p = 0.50), suggesting that the overall disease severity was not very different between groups. After excluding patients who stayed only one night in the hospital, 117 patients (48 without and 69 with the system) were included in the analysis. Without the system, average daily visit counts were 16.3 [5.5 – 20.3] times in total and visits for vital sign measurements were as follows; visits for body temperature 2.8 [2.3 – 3.0] times, respiratory rate 2.6 [2.0 – 3.0] times, peripheral oxygen saturation (SpO2) 2.7 [2.0 – 3.0] times, heart rate 2.7 [2.2 – 3.0] times, and blood pressure 2.7 [2.3 – 3.0] times per day. With the system, the numbers of these visits were significantly smaller (Figure 2); total visit counts 7.5 [4.5 – 17.5] times (p = 0.009 vs. without the system), body temperature 1.4 [0.0 – 2.3] times (p <0.001), respiratory rate 1.4 [0.2 – 2.0] times (p <0.001), SpO2 1.4 [0.0 – 2.3] times (p <0.001), heart rate 1.4 [0.0 – 2.0] times (p <0.001), blood pressure 1.6 [0.1 – 2.2] times (p <0.001). In a multivariable linear regression model, the association of the use of the system with the number of patient visits was significant even after being adjusted with the hospital length (coefficient 3.5, $p = 0.026$).

Among 117 patients, 91 (77.8%) were from two hospitals (48 from hospital A, 43 from B, and 26 from two other hospitals). The number of patient visits in these hospitals was significantly different (5 [4 – 5] in A, 19 [18 – 24] in B, and 12 [9 – 17] in others, $p < 0.001$). Importantly, in both hospitals A and B, the numbers decreased with the system (median 5.0 to 4.3 in A and 21.0 to 18.6 in B, $p = 0.037$ and 0.025, respectively). The lengths of stay in each hospital were not significantly different ($p = 0.40$) among hospitals, suggesting that the difference in the number of patient visits was not due to the severity in each hospital.

***Mental status***

In the five institutes, 101 nurses answered the questionnaire in total. One nurse was excluded from the analysis due to insufficient answers. Overall, the nurses' impression was favorable. The majority of nurses agreed that the system is easy to use (87.1%), reduces their work burden (75.2%), and was effective for reducing healthcare providers' infection risk in hospitals (79.1%) and in nursing accommodations (95.0%). Moreover, 74.3% of nurses answered that the system made them feel relieved. Nurses' distress and depression assessed using IES-R and PHQ-9 were at the minimum even before the system was introduced (IES-R 10 [5–27]; above 22 is considered "a concern" and above 33 is the optimal cutoff for posttraumatic stress disorder; PHQ-9 3 [1–8]; 0–4 is considered minimum or no depression). Their scores did not worsen after the system was put in place (IES-R 11 [3–27], p = 0.72 and PHQ-9 3 [1–9], p = 0.57) (Figure 3).

## Discussion

In the present study, we found that the telemedicine system is associated with healthcare providers having to pay significantly fewer visits to COVID-19 patients. Specifically, visits for vital sign measurements were dramatically fewer with the said telemedicine system. It was also found that a majority of the nurses who participated in the study had a favorable impression toward the system. However, we did not see a significant difference in the nurses' mental health status before and after the system was introduced. To the best of our knowledge, this is the first study that showed scientific evidence of telemedicine's clinical impact on the reduction of physical contact between COVID-19 patients and healthcare providers.

One of COVID-19's most devastating aspects is that it can be fatal even among young people who do not have chronic comorbidities (2, 18). Moreover, several persistent symptoms have been reported as "long COVID" even among those who have already recovered (19). Thus, the most important mission in medical facilities during the COVID-19 pandemic is to protect healthcare providers from infection risk. Reducing patient room visits can be effective in reducing the risks because this disease spreads mainly through physical contact and respiratory droplets. Vital sign measurement requires physical contact with the patient; thus, there is an inherent risk of infection. In the present study, the nurses were not recommended nor forced to avoid patient visits. However, the nurses skipped patient room visits because they felt that these were unnecessary given that the system allowed them to check patients' self-measured vital signs. These measurements have been reported as accurate in a previous study (15). As a result, patient visits, especially those for vital sign measurements, decreased dramatically ($p < 0.001$ for all vital signs). No nurse felt that the system was dangerous or increased the risk of disease deterioration. The intra-institutional analysis showed that the patient visits were decreased in both the hospital with the least patient visits and the one with the most visits, suggesting that the system may be effective regardless of the base number of patient visits.

The issues regarding disposal of PPEs during the COVID-19 pandemic is another concern that may be mitigated by our system. Donning and doffing a PPE for every patient visit takes a significant amount of extra time and increases healthcare providers' work burden. Moreover, many hospitals around the world experienced critical supply shortages of PPEs due to high demand (20). Therefore, less frequent patient room visits are not only effective in reducing healthcare providers' work burden, but also in saving resources such as PPEs.

A possible bigger problem than the actual infection risk is the mental stress that is caused by healthcare providers' infection risks and increased work burden. There have been reports on healthcare providers developing depression and not being able to continue caring for COVID-19 patients (21, 22). In our study, many nurses (74–95%) had a favorable impression toward the system. Notably, 74.3% of the nurses responded that the system helped them feel relieved. IES-R, a questionnaire for assessing subjective distress caused by traumatic events, and PHQ-9, a major depression module, showed no major distress or depression among healthcare providers even before the system was introduced in the hospitals. Moreover, the results were not different even with the system in place. We consider these results as favorable. The first period of the study (i.e., before the system was introduced) was just after Japan's second wave of the COVID-19 pandemic, and the number of daily new cases was relatively suppressed (Figure 4). However, at the time when the second questionnaire was administered (i.e., with the system), Japan was in the middle of its third wave of the pandemic. At this time, the patient load on hospitals and social tension were at their highest. There might be a possibility that the telemedicine system partly protected the nurses from developing distress and depression symptoms. However, this is only our speculation, and further studies including randomized trials are warranted.

### *Limitations*

This study has several limitations. First, we did not collect detailed information from patients due to the study's nature that focuses on healthcare providers. It is possible that patients' backgrounds and disease severity varied before and after the telemedicine system was introduced. However, the length of hospital stay was not different between the two periods. Additionally, the patients who required an intensive care unit stay and could not personally measure their vital signs were excluded from the study. On the other hand, we excluded the accommodation that serves to less severe patients who do not require in-hospital care stay. Our present results may not be applicable for such facilities where patients with mild symptoms stay. Thus, the present study only included patients with mild symptoms, most of whom did not require oxygenation. We did not track the nurses' mental health continuously; thus, the mental health might change between the first and the second questionnaires. As discussed, however, we speculate that their mental health should not have improved in this period since the COVID-19 circumstances were getting worse in this period; the possibility of this bias inappropriately overestimated the system was low. Moreover, the pre-post quasi-experimental design of this study might introduce nonneglectable confounding factors. For example, the nurses might have increased workload that reduced their ability to frequently visit each patient. The possibility of these potential confounders should be appropriately acknowledged, and further studies with more rigorous designs are warranted in the future. Next, although the five questions showed the nurses' positive impression of the system, it should be appropriately acknowledged that these questions have not been validated. Finally, we did not assess the actual risk of healthcare providers developing COVID-19 because the event rate would have been very low (23). Larger studies in the future may investigate if the system truly decreases healthcare providers' infection risk.

## Conclusions

This study found that a telemedicine system for the patients' self-assessment of their vital signs was associated with healthcare providers having to pay fewer patient visits, especially for vital sign measurements. A majority of the nurses felt that the system was effective and made them feel relieved. Therefore, this system can be used as an effective alternative to traditional vital sign measurement by healthcare providers, possibly reducing their infection risk.

**Acknowledgements**

The authors thank all nurses and the medical staffs who face and fight against this COVID-19 pandemic at Juntendo University Hospital, Juntendo University Nerima Hospital, Fujisawa City Hospital, Tokyo-Kita Medical Center, and Shonan Village Center. The authors thank them for their dedication in caring for patients and their excellent contributions during data acquisition.

**Funding Statement**

This research was supported by Japan Agency for Medical Research and Development (AMED) under Grant Number JP20he0522003, and by Japan Society for the Promotion of Science (JSPS) KAKENHI (Grant Number, 21K08116).

**Competing Interests Statement**

Dr. Kagiyama, Dr. Daida, and Dr. Kasai are affiliated with a department funded by Philips Healthcare, Asahi KASEI Corporation, Inter Reha Co., Ltd., and Toho Holdings Co., Ltd.

based on collaborative research agreements. The other authors declare no conflict of interest.

## Contributorship Statement

NK; planning, data acquisition, statistical analysis, and writing

T. Komatsu; data acquisition, and critical reviewing

MN; data acquisition, and critical reviewing

MH; data acquisition, and critical reviewing

MK; data acquisition, and critical reviewing

WM; data acquisition, and critical reviewing

HD; data acquisition, and critical reviewing

TM; data acquisition, and critical reviewing

TN; data acquisition, and critical reviewing

MS; data acquisition, and critical reviewing

KM; data acquisition, and critical reviewing

HA; supervision, and critical reviewing

T. Kasai; planning, supervision, and critical reviewing

## Data Availability statement

Research data are not available for public access due to patient privacy concerns but can be obtained from the corresponding author on reasonable request approved by the institutional review boards of all participating institutions.

## Figure Legends

**Figure 1. Overall pipeline of the telemedicine system**

The system semi-automatically uploads the data to secured cloud storage. The digital manometer automatically then transfers the data to the gateway via Bluetooth. The other parameters, body temperature, and $SpO_2$ are manually transferred to the gateway by holding the thermometer and pulse oximeter to the gateway. A mat-type air pressure sensor placed under the bed mattress automatically and continuously measures the patients' respiratory rates. These measurements can be looked into by healthcare providers from clean zones without the risk of infection.

**Figure 2. Times of room visits without and with the system**

Vital sign-specific visits and total patient visits were significantly fewer with the system. (BT: body temperature; RR: respiratory rate; $SpO_2$: peripheral oxygen saturation; HR: heart rate; BP: blood pressure)

**Figure 3. Change in mental status**

No significant changes were observed in mental status, without and with the system.

**Figure 4. The number of newly diagnosed COVID-19 cases in Japan**

The system was introduced to the hospitals just after Japan's second wave of the COVID-19 pandemic. The number of daily new cases was relatively suppressed with the system. The pandemic's third wave occurred right after the system's introduction, and social tension rose to the maximum. The questionnaires were assessed just before and soon after the 'with the system period' finished.

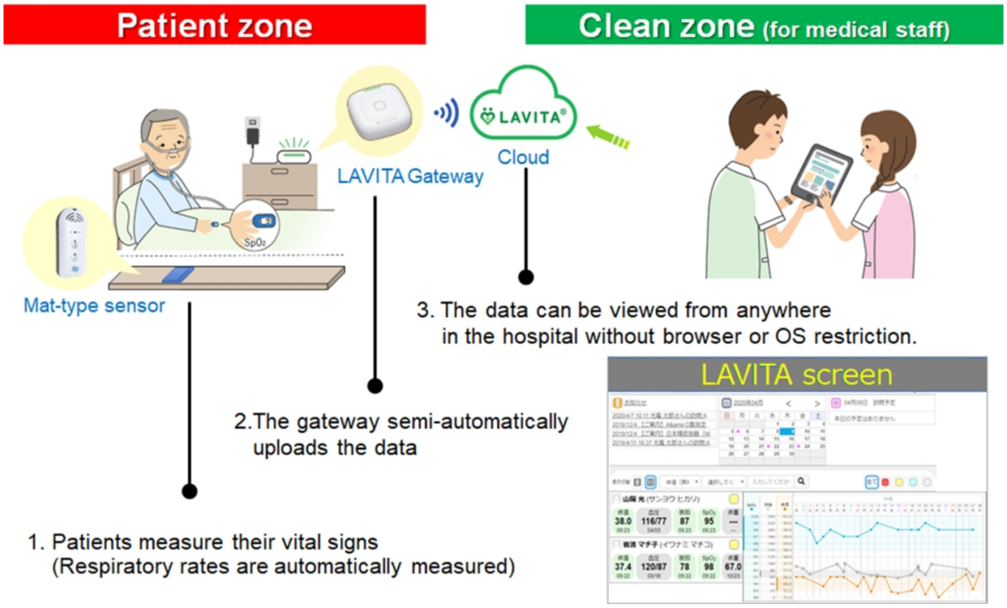

Figure 1. Overall pipeline of the telemedicine system
The system semi-automatically uploads the data to secured cloud storage. The digital manometer automatically then transfers the data to the gateway via Bluetooth. The other parameters, body temperature, and SpO2 are manually transferred to the gateway by holding the thermometer and pulse oximeter to the gateway. A mat-type air pressure sensor placed under the bed mattress automatically and continuously measures the patients' respiratory rates. These measurements can be looked into by healthcare providers from clean zones without the risk of infection.

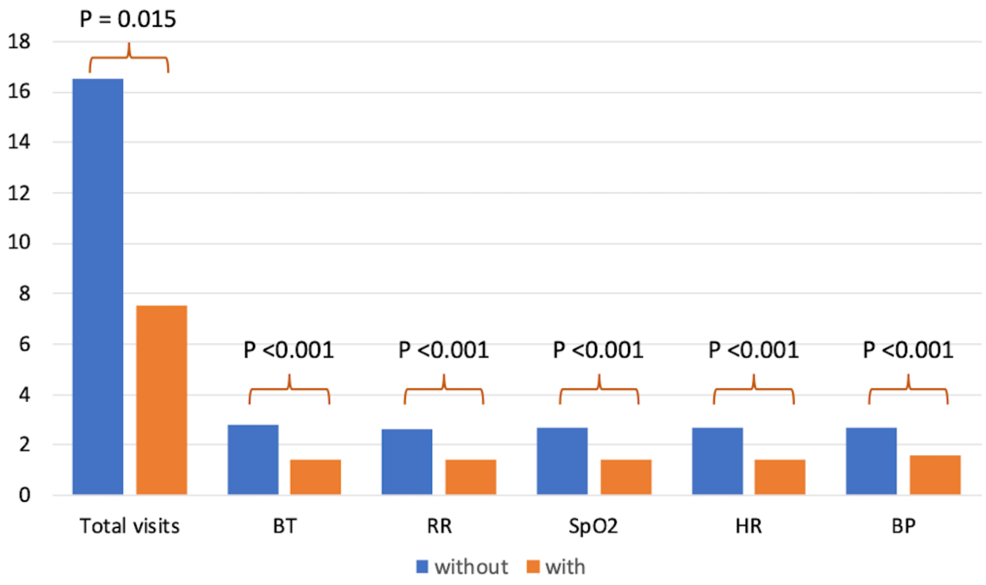

Figure 2. Times of room visits without and with the system
Vital sign-specific visits and total patient visits were significantly fewer with the system.
(BT: body temperature; RR: respiratory rate; SpO2: peripheral oxygen saturation; HR: heart rate; BP: blood pressure)

99x59mm (300 x 300 DPI)

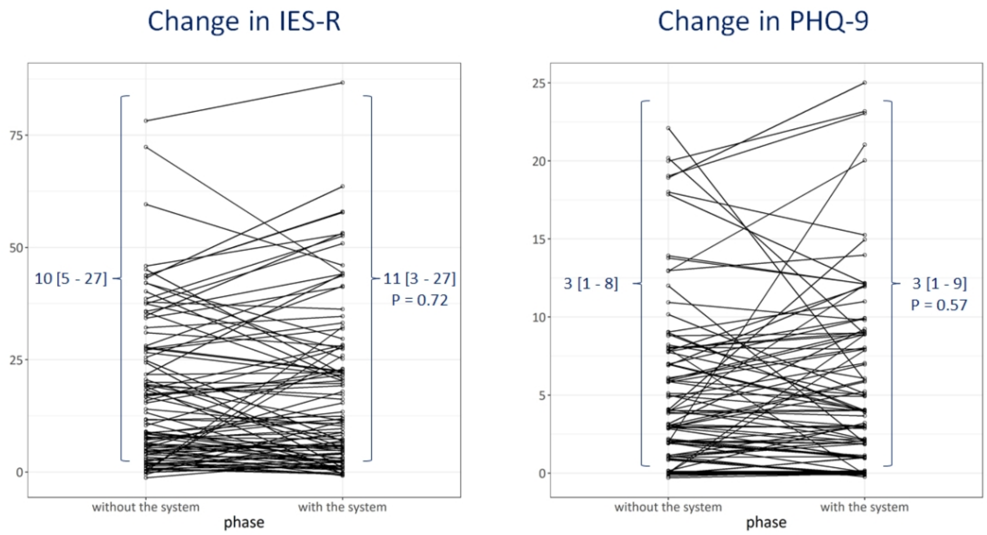

Figure 3. Change in mental status
No significant changes were observed in mental status, without and with the system.

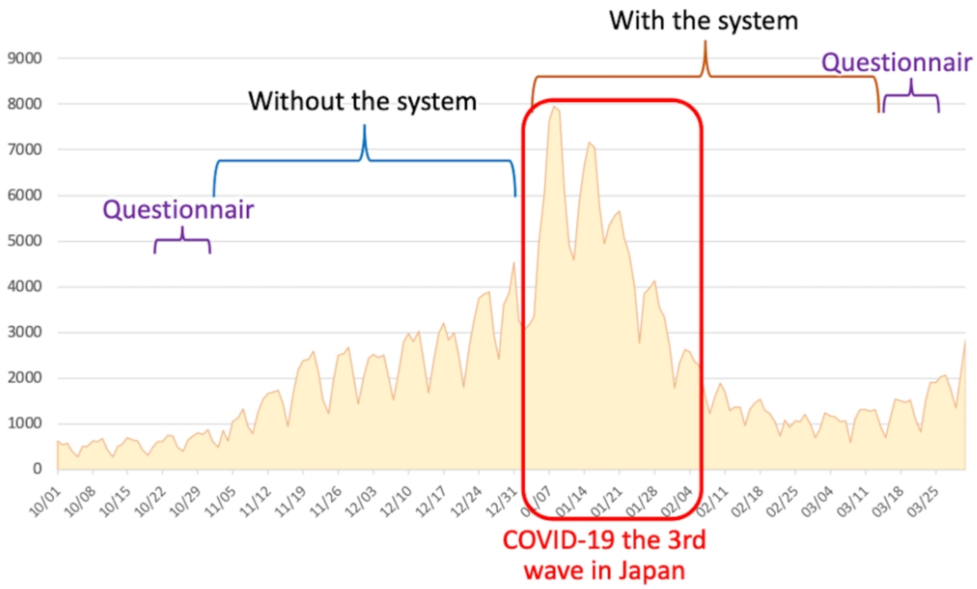

Figure 4. The number of newly diagnosed COVID-19 cases in Japan
The system was introduced to the hospitals just after Japan's second wave of the COVID-19 pandemic. The number of daily new cases was relatively suppressed with the system. The pandemic's third wave occurred right after the system's introduction, and social tension rose to the maximum.

99x61mm (300 x 300 DPI)
